# The Perils of Pregnancy: A Case Report of Subglottic Stenosis​

**DOI:** 10.7759/cureus.76525

**Published:** 2024-12-28

**Authors:** Carlos A Saldarriaga, Bryan Choi

**Affiliations:** 1 Emergency Department, Bayhealth Hospital, Dover, USA

**Keywords:** acquired subglottic stenosis, endoscopic laser ablation, idiopathic subglottic stenosis, pregnancy-related complications, subglottic stenosis

## Abstract

Subglottic stenosis (SGS) presents a rare, yet challenging condition characterized by airway obstruction below the glottis, with diverse etiologies ranging from congenital to acquired factors like intubation or autoimmune diseases. Diagnosis and management of SGS during pregnancy are particularly complex due to limited literature and diagnostic consensus. This article presents a case of a 26-year-old pregnant woman presenting with escalating dyspnea and stridor attributed to SGS, most likely secondary to idiopathic etiology. Initial assessments, including a CT scan and fiberoptic laryngoscopy, confirmed the diagnosis of SGS showing a narrow subglottic trachea. Given the complexity of the case, she was transferred to a tertiary care center where she underwent CO2 laser excision, balloon dilation, and submucosal Triamcinolone injection. Pre and postoperatively, the patient was managed with corticosteroids, antibiotics, and bronchodilators. Her condition improved significantly, as evidenced by a follow-up strobovideolaryngoscopy on day 15, which showed a symmetric vocal fold, adequate vibratory motion, and widely patent subglottic larynx. This report emphasizes the importance of tailored, multidisciplinary management of SGS during pregnancy, with endoscopic resection and adjuvant therapies proving to be effective interventions. Regular follow-up is crucial due to the potential for recurrence within three years post-treatment.

## Introduction

Subglottic stenosis (SGS) is characterized by the obstruction of the central airway, specifically in the region below the glottis and superior to the first two tracheal rings. SGS is a rare condition [1] and the presenting symptoms can be confusing. SGS can be either congenital or acquired. Acquired causes encompass granulomatosis with polyangiitis, sarcoidosis, gastro-esophageal reflux disease, or prior prolonged intubation or tracheostomy. Up to 20% of patients have primary idiopathic subglottic stenosis (iSGS) [2], which is diagnosed after all other causes are ruled out [3].

SGS is rarely encountered during pregnancy; thus, most of the existing literature is based on a limited number of case reports. Consequently, there is no consensus on adequate diagnostic tools and therapeutic interventions [3].

In this report, we present a case of a pregnant woman who was in her second trimester and presented to the Emergency Department with iSGS. We describe her multidisciplinary management and the subsequent outcomes.

## Case presentation

**Figure 1:**
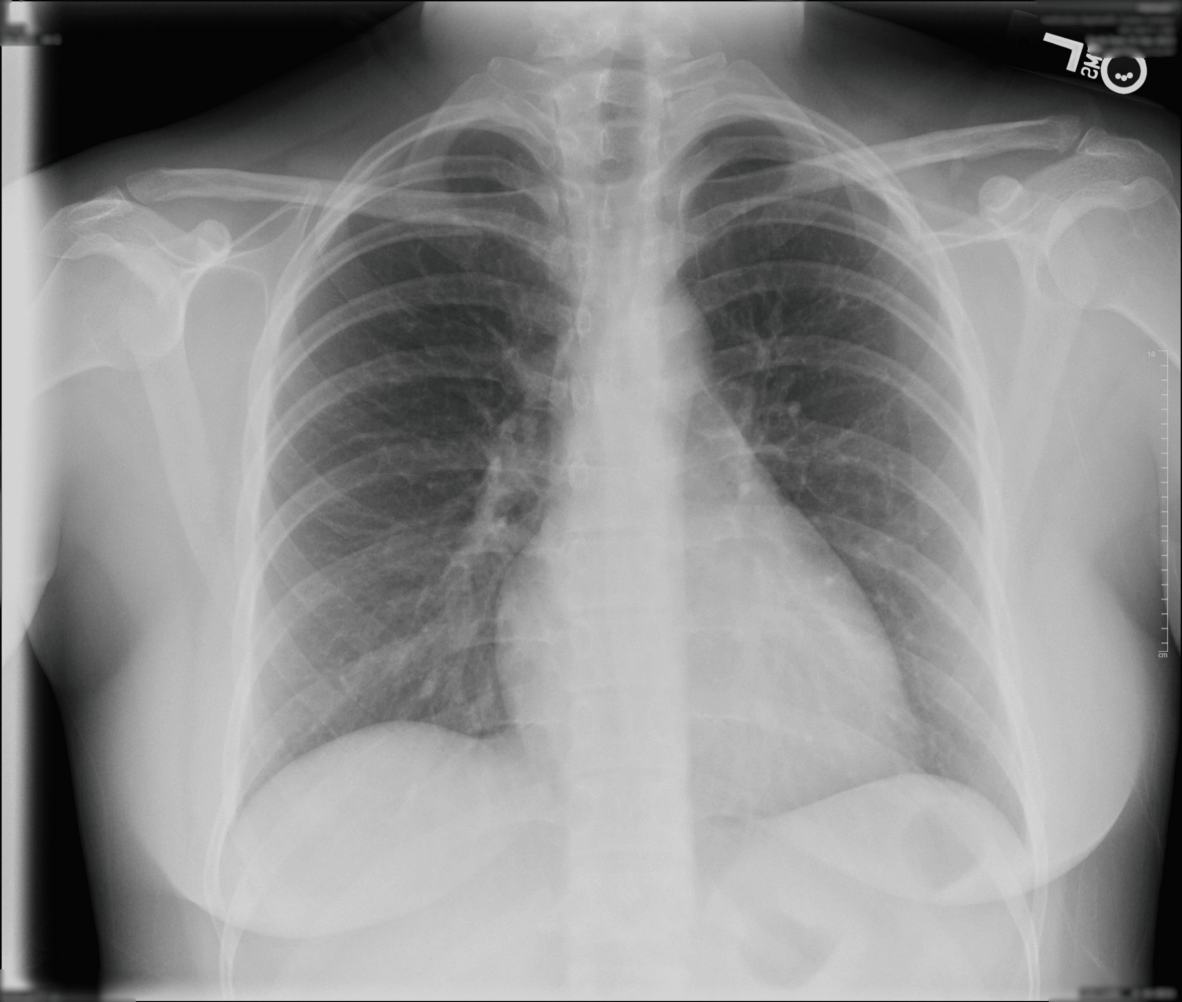
Normal chest X-rays

**Figure 2:**
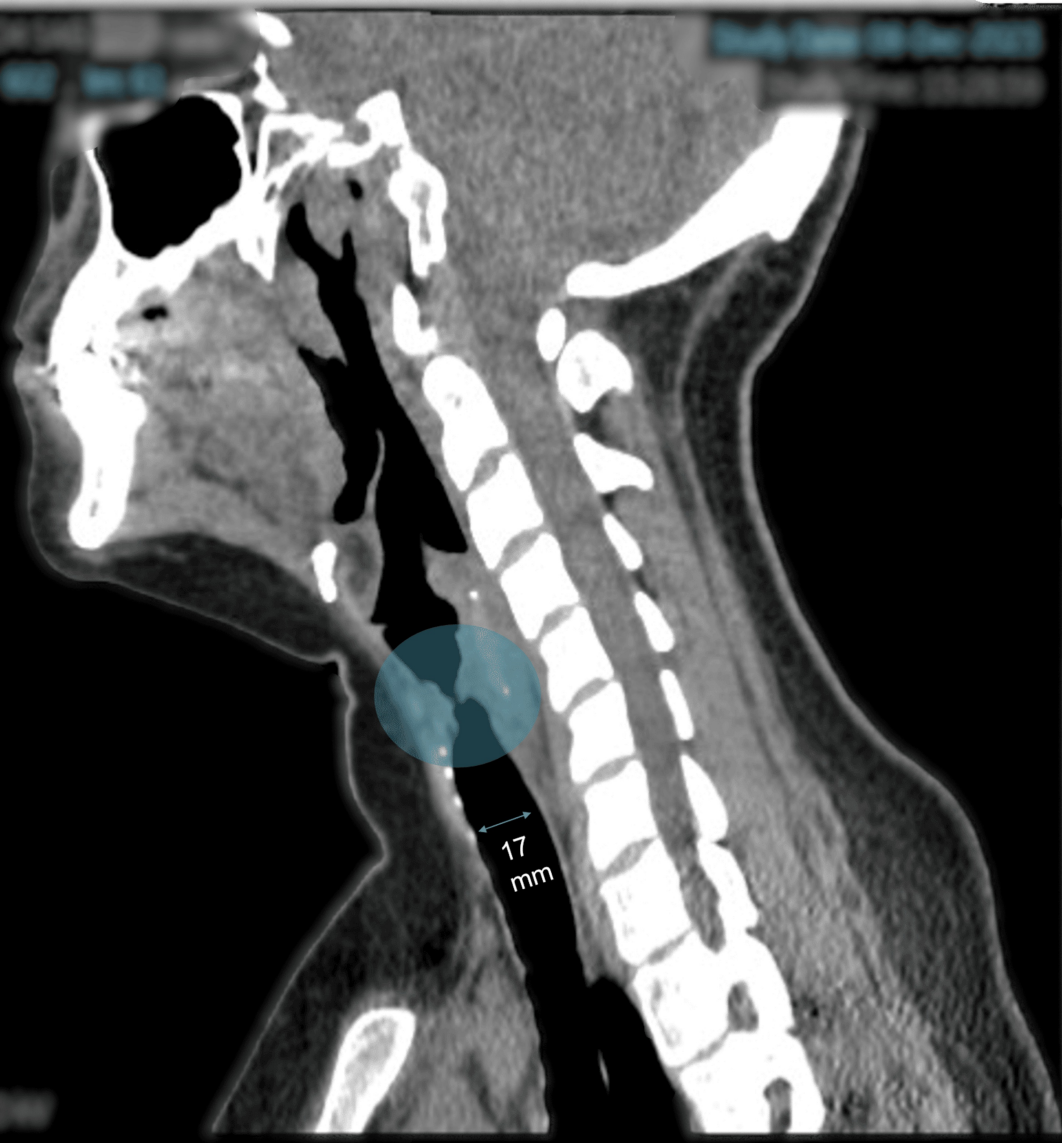
Mucosal thickening of the subglottic trachea along the margins with a narrowed caliber

A 26-year-old woman, G2P1V1, 17 weeks pregnant, was admitted to our community teaching hospital’s Emergency Department following three weeks of escalating dyspnea and stridor. Her medical history included herpes simplex virus and hypertension in a previous pregnancy. She has no previous surgical history. During the initial assessment, the patient exhibited biphasic stridor, most audible over the cricoid and discernible from several feet away. Her physical examination revealed the following vitals: blood pressure: 125/54, heart rate: 86, respiratory rate: 18, oral temperature: 36.8 °C (98.2 °F), and oxygen saturation: 99% on room air. She appeared comfortable with no signs of respiratory distress. We ordered a comprehensive metabolic panel and complete blood count with differential that revealed normal values. COVID-19, RSV, and Influenza PCR were negative. The Strep A rapid test was negative. Chest X-ray showed no evidence of overt pneumonia (Figure 1). A CT scan of the neck without intravenous contrast revealed mucosal thickening of the subglottic trachea along the margins with a narrowed caliber measuring 6 mm medial to lateral, suggesting SGS (Figure 2).

Flexible fiberoptic laryngoscopy revealed normal laryngopharynx anatomy with normal vocal cord mobility. The patient was started on albuterol 90 mcg (two puffs every six hours) and a single dose of methylprednisolone 125 mg.

Informed consent was obtained from the patient to perform the CT scan and fiberoptic laryngoscopy. Given the complexity of the case, the patient was transferred to Thomas Jefferson University Hospital. She was started on azithromycin 500 mg (every 24 hours), mometasone 220 mcg (two puffs every 12 hours), and prednisone 100 mg (every 24 hours). The patient underwent microscopic direct laryngoscopy that showed membranous SGS narrowing the airway to approximately 30-40% of normal diameter. The stenotic region was excised using a CO2 laser. After the laser ablation was completed, the larynx was dilated under direct visualization. A 12 mm Aeris balloon was placed and inflated to maximal dilation for three separate intervals of 8-10 seconds. This was repeated with a 14 mm Aeris balloon with similar intervals of dilation. After completion of dilation with a 14 mm balloon, the airway appeared normal in diameter. Triamcinolone (40 milligrams) was injected submucosally in the subglottic larynx, followed by laryngeal dilation with Aeris balloons. There were no complications during or immediately after surgery. At the time of her discharge, she was alert, and her voice was clear and strong. She was prescribed an 18-day course of prednisone 100 mg, azithromycin 500 mg, and albuterol, with follow-up appointments planned with ENT and OB/GYN.

**Figure 3:**
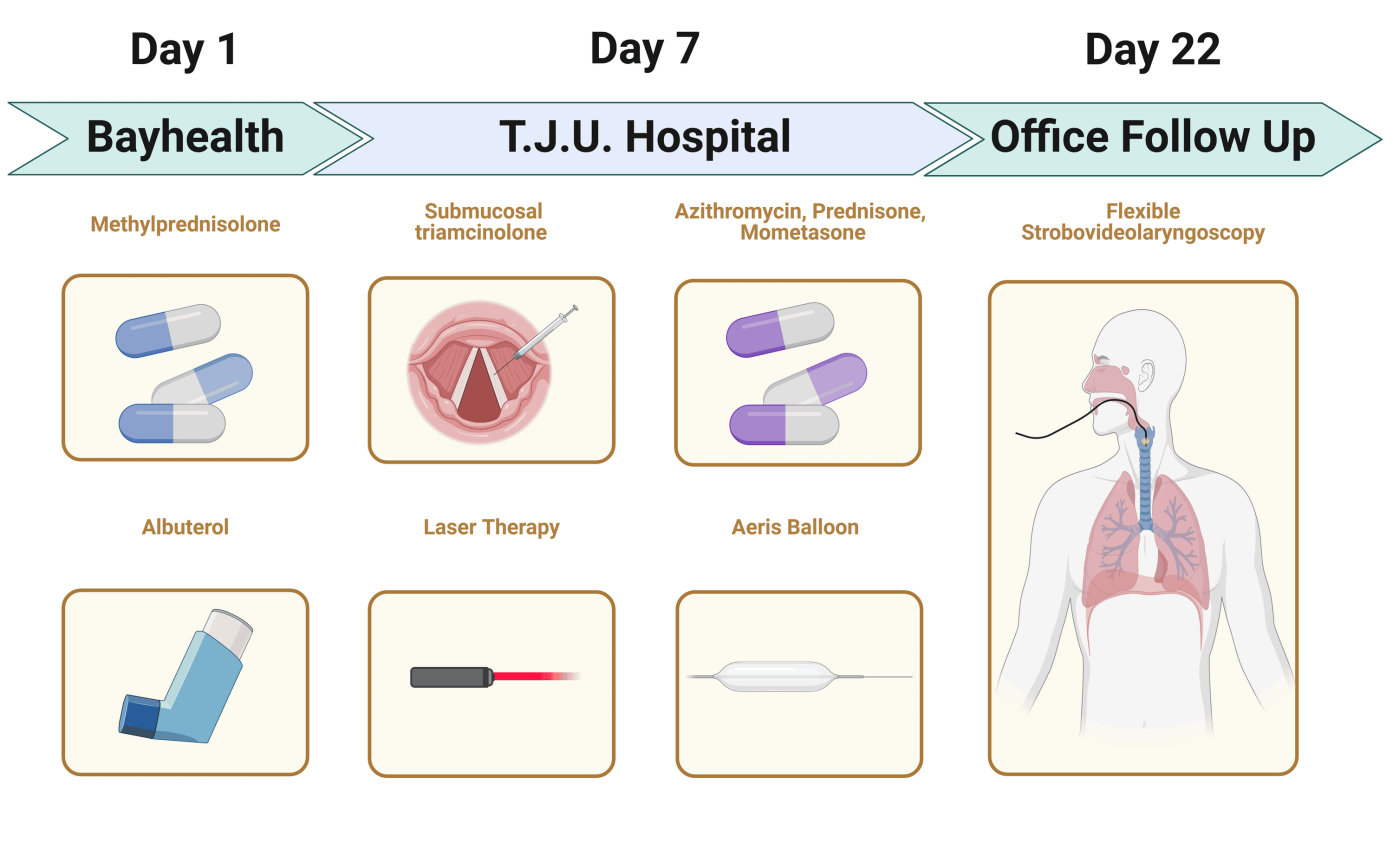
Diagnostic and therapeutic timeline

The patient spent three days in the hospital, during which she was evaluated by multiple consulting services including OB/GYN, ENT, and internal medicine. The patient underwent a flexible strobovideolaryngoscopy on day 15, which showed normal and symmetric vocal fold and vibratory motion. There was a small amount of mucus anteriorly in the subglottis; otherwise, the subglottic larynx was widely patent. The patient did not show any signs of complications 15, 21, and 124 days after surgery (Figure 3).

## Discussion

SGS is a rare condition and the incidence and prevalence in pregnancy are unknown. This case adds valuable data to the limited pool of information available on this condition. In addition, outlines a successful diagnosis and treatment pathway for this disease, demonstrating the use of various diagnostic tools and therapeutic interventions.

Most of the SGS cases are most commonly acquired after tracheal intubation [4]. Some of the factors that increase the risk of SGS are multiple or traumatic intubations, inappropriate endotracheal tube size [5], unplanned extubation, or prolonged intubation [6,7]. iSGS causes up to 80% of SGS [8], as in our patient with no identifiable risk factors. Although the pathophysiology of iSGS is unclear, estrogen, IL-17A/IL-23 axis, and gastroesophageal disease may play a role. Most of the iSGS cases are reported in women [9,10] and symptoms seem to be worse during pregnancy [11]. Indeed, recent studies show an increased number of estrogen and progesterone receptors in the stenotic tissue of iSGS compared to either normal tissue or posttraumatic SGS [12-15]. In addition, tracheal mucosa of iSGS patients upregulates IL-23/IL-17A compared to control healthy trachea [16]. Different studies show that IL-17A directly stimulates the proliferation of iSGS scar fibroblasts, enhances extracellular matrix production, and intensifies local inflammatory signaling [17]. Lastly, close to half of patients with iSGS were found to have gastroesophageal reflux disease by esophageal pH impedance testing [18]. However, further studies are needed to determine if GERD causes iSGS.

Flexible nasopharyngoscopy is commonly used for the definitive diagnosis of SGS [19-22]. However, a CT scan and MRI of the neck can be used to measure the length of stenosis [19-21]. Treatment options include endoscopic dilation, endoscopic resection with adjuvant medical therapy, and open cricotracheal resection. Of note, iSGS is a recurrent disease. About 22.8% of patients have a recurrent surgical procedure during the following three years after the initial procedure [2]. During endoscopic dilation, a balloon is used to expand the stenotic segment. Around 28% of patients will require a new surgical procedure during the next three years [2]. Some of the risks of this procedure include temporary tongue paresthesia, transient postoperative subcutaneous emphysema, and dental injury [2]. Death secondary to airway obstruction after the procedure is rare [2]. Endoscopic resection with adjuvant medical therapy uses a carbon dioxide laser to resect the stenotic area with or without direct injection of steroids and Mitomycin-C [23-25]. This is followed by long-term adjuvant medication including inhaled corticosteroids, proton pump inhibitors, and/or antibiotics [1]. Around 12.4% of patients will require a new surgical procedure during the next three years after endoscopic resection with adjuvant medical therapy. This therapy modality has a similar perioperative adverse event profile compared with endoscopic dilation; however, adverse reactions to antibiotics are common [2]. Open cricotracheal resection involves the removal of the stenotic portions followed by anastomosis of the proximal and distal tracheal sections. 1 % of patients will require a new surgical procedure during the next three years. However, about 10% of patients require a temporary tracheostomy, unplanned return to the operating room during their initial hospitalization, and permanent unilateral vocal fold paralysis [2]. Given the above, endoscopic resection with adjuvant medical therapy has become a popular treatment choice.

This case is significant for several reasons. It describes a rare instance of a 26-year-old pregnant woman presenting with SGS. It outlines a successful diagnosis and treatment pathway for this disease, demonstrating the use of various diagnostic tools and therapeutic interventions. The patient’s condition improved post-treatment. The case introduces a novel treatment method involving CO2 laser excision, laryngeal dilation with Aeris balloons, and submucosal injection of triamcinolone. This approach treated the SGS effectively and ensured the patient’s safety and comfort.

Differential diagnoses were excluded based on the patient’s symptoms, age, and test results. Granulomatosis with polyangiitis was not strongly suspected due to over 80% of patients with granulomatosis with polyangiitis being present with sinusitis and lung disease. Other common features that were absent in this case include nasal involvement, joint disease, fever, cough, skin symptoms, otitis media, and hearing loss [26]. Furthermore, the course of untreated Wegener's granulomatosis is rapid, with up to 90% mortality within five months [27], indicating that the patient would likely have been deceased without treatment. Croup was ruled out as it typically presents with a “barking” cough, stridor, and hoarseness and is more common in children. Subglottic cysts and tracheal rings were excluded as these would typically be visible on a CT scan or laryngoscopy, but only mucosal thickening of the subglottic trachea was observed. Subglottic hemangioma was ruled out as it would appear as a mass on imaging, and the CT scan did not reveal any mass. Vocal cord paralysis was excluded as this condition would typically present with voice changes, such as hoarseness, rather than stridor. Also, the flexible nasopharyngoscopy showed normal vocal fold and vibratory motion. Therefore, iSGS was strongly suspected.

## Conclusions

This case highlights the successful diagnosis and management of iSGS in a pregnant patient, utilizing a multidisciplinary approach and novel treatment involving CO2 laser excision, balloon dilation, and submucosal steroid injection. It underscores the importance of considering SGS in pregnant patients presenting with respiratory symptoms and the effectiveness of combined endoscopic techniques for treating this condition. Clinicians should be aware of the potential for SGS in similar presentations and consider flexible nasopharyngoscopy and advanced imaging for accurate diagnosis. For treatment, endoscopic resection with adjuvant therapy offers a balance of efficacy and safety, with careful monitoring and follow-up to manage recurrence risks.
